# The mobilisation of professional identity: A scoping and lexical review

**DOI:** 10.1371/journal.pone.0298423

**Published:** 2024-04-16

**Authors:** Ann Dadich, Stephanie Best

**Affiliations:** 1 School of Business, Western Sydney University, Parramatta, NSW, Australia; 2 Peter MacCallum Cancer Centre, Melbourne, VIC, Australia; Federal University of Goias: Universidade Federal de Goias, BRAZIL

## Abstract

Interprofessional care obliges different healthcare professions to share decision-making and sometimes, practices. Given established hierarchies, it can be difficult to promote interprofessional care, partly because of the need to reshape professional identities. Despite interest in effective interprofessional care, there is limited research on how professional identity can be mobilised to promote it. A scoping review as well as lexical review of academic publications was conducted to address this void. After searching seven academic databases and screening the identified publications, 22 publications met the inclusion criteria. They collectively reported on 22 interventions, most of which were used in healthcare. The scoping review suggested there is some evidence that professional identities can be mobilised. Yet, of the 22 interventions, only ten explicitly targeted professional identity. The most common intervention was a training or development program, followed by workplace redesign. The need for internal motivation to mobilise professional identity was reported as was the impact of external drivers, like extending the scope of practice. Extending these findings, the lexical review demonstrated that, among the 22 publications, the relationship between professional identity and mobilisation did not feature prominently within the discourse. Furthermore, it seems that geography matters–that is, while all the publications spoke of professional identity, they differed by region on how they did this. Given these findings, concentrated scholarship is needed on the relationship between professional identity and interprofessional care, lest interprofessional care programs have limited, sustained effect. Implications for scholars and practitioners are explicated.

## Introduction

Worldwide, interprofessional care represents best practice [1–3]. It involves ‘multiple health workers from different professional backgrounds [who] provide comprehensive services by working with patients, their families, carers and communities to deliver the highest quality of care across settings’ [4]. Interprofessional care is important for at least three key reasons. First, given the rise of chronic and complex illnesses [5, 6], patients and their carers often require different forms of healthcare from different professions at different times–as such, interprofessional care can offer patients and carers timely access to appropriate healthcare. Second, interprofessional care can aid effective and efficient communication between different professions [7], thereby averting duplicative effort or, worse still, the possibility that patient and carer needs remain overlooked. Third (and relatedly), interprofessional care can offer economic benefits [8], which is particularly important for resource-limited health services [9–11].

Despite the importance of interprofessional care, it does not always occur. In addition to educational, organisational, and policy-related challenges [12–14], this is partly due to professional identity–that is, ‘an organized group’s norms, values, and behavioral knowledge that situate an individual into group membership’ [15]. Professional identity can be robust and near-impermeable [16]. This resistance in turn can hinder how those who represent different professions work together to deliver quality patient care and promote patient wellbeing [17, 18]. This might account for research on and calls for research on identity mobilisation [19–21].

Building on extant research, the aim of this study is to clarify whether the professional identity of established professions can be actively mobilised–that is, changed or reshaped. The review purposely focuses on established professions, rather than students, given that students’ professional identity is typically developing and perhaps more amenable to change [22, 23]. The study aim is achieved via the complementary methods of a scoping review and a lexical review. While the former served to scope a body of literature [24, 25], the latter was used to diagrammatically map how discourse within the identified publications travelled together [26].

This article is structured as follows. It commences with an overview of the relationship between professional identity and mobilisation. It then describes and justifies how the reviews were conducted and the associated results. Following this, the article summarises the key findings and clarifies the implications for scholars as well as those who manage and deliver healthcare.

### Professional identity and mobilisation

Professional identity can be described as, ‘the relationship between the collective level of the profession and the individual level of the professional’ [27]. The tertiary education of healthcare professionals not only qualifies a practitioner to practice in their field, but imparts a sense of identity with their profession, along with the responsibilities of being a health professional [28]. Learners are influenced by their teachers to develop their professional identity through enculturation and situated group learning environment–‘Situating their learning in groups allows each individual to move from peripheral participation to active engagement’ [29]. Both enculturation and situated group learning environment form part of the formal and explicit curriculum, as well as the informal and hidden curriculum [30, 31]. In additional to tertiary education, professional identity is influenced by myriad factors, including: gender; cultural background; professional ethos; as well as working experience [32–35].

As the health systems of many Western nations face contemporary social and economic challenges [36, 37], healthcare professions (*sensu lato*) are changing–so too are expectations of what and how they deliver care [38]. This includes increasing calls for interprofessional healthcare. This can have considerable implications for professional identity as professionals shape, and are shaped by each other [39]. Consider for instance, the behavioural effects of modelling ethical decision-making and behaviour [40]–in their examination of the dual identities of American army medics (as doctors and soldiers), Leavitt and colleagues [41] found moral duty to be entrenched within a professional’s identity, shaping their medical and military practices. Yet the challenge is the relationship between professional and team identities. As Davoli and Fine [42] observed:

When working with professionals in the same field, it is easy to become complacent about our identity. Everyone assumes familiarity with the field, and there is little or no need to defend one’s position. However, working among professionals from other fields requires everyone to be comfortable with his or her roles and associated goals in his or her chosen profession… It is important that professional identities not become watered down while providing collective services.

Although the development of professional identity is iterative [27], the extent to which it can be purposely prepared for interprofessional healthcare using an intervention is less clear. With the increasing calls for interprofessional healthcare, the ability to mobilise or flex professional identities might prove useful. This reflects the case that others have proffered about the need to consider whether and how professional identity can be mobilised [43]. For instance, following their critical scoping review on the social identity approach, Kreindler and colleagues [19] suggested that addressing professional silos requires a consideration of how the identities that matter most to people can be mobilised. Similarly, after scoping the literature on interprofessional care and professional identity, Best and Williams [20] argued that professional identity should be mobilised to accommodate a changing workforce–moreover, such mobility requires ‘active engagement and management and cannot be ignored, particularly during periods of uncertainty’. Further still, in their qualitative study, Harvey and colleagues [21] concluded that mobilising facets of professional identity can enable staff members to achieve their managerial objectives. Accordingly, scholars have called for a greater understanding of how employees manage ‘working across professional and organisational boundaries’ [44]. This was the impetus for a review to ascertain the interventions that mobilise professional identity.

## Materials and methods

### Scoping review

Guided by literature on the conduct of scoping reviews [45, 46], a protocol was developed and registered with the Open Science Framework (osf.io/g69db). First, the overarching research question was articulated–namely, can the professional identity of established professions be actively mobilised? Second, a search strategy was developed and tested to source publications from the academic database, Business Source Complete (EBSCO, see Table 1), irrespective of publication date. Following this, the search strategy was deployed via six additional academic databases, irrespective of publication date–these included: CINAHL Plus with Full Text (EBSCO); Health Business Elite (EBSCO); Health Source: Nursing/Academic Edition (EBSCO); MEDLINE (Ovid); APA PsycArticles (EBSCO); and APA PsycInfo (EBSCO). These were searched on June 25, 2023 via the following strategy to capture publications pertaining to the mobilisation of professional identity:

1. “Dual identity” or “professional identit*” or “professional self concept” or “professional self-concept” or “professionals’ self-concept” or “inter-disc* identity” or “interdisc* identity” or “inter-profess* identity” or “interprofess* identity” or “multi-disc* identity” or “multidisc* identity” or “multi-profess* identity” or “multiprofess* identity” or “trans-disc* identity” or “transdisc* identity” AND
2. Checklist* or course* or educat* or guid* or intervention* or lesson* or model* or module* or prevent* or program* or “professional development” or resource* or strateg* or train* or workshop*

These terms were sought within publication titles or abstracts to identify publications that expressly focused on initiatives that served to mobilise professional identity. Although potentially limiting, alternative approaches largely served to identify irrelevant publications–this might be partly due to the multifaceted nature of professional identity [47]. As such, a focused and strategic search strategy was used. A total of 2,926 scholarly publications in the English language were identified. Of these, 1,041 were duplicates and were thus removed, leaving 1,885 publications.

**Table 1:** Business Source Complete (EBSCO) search.

#	Searches	Results
1	TI (“Dual identity” or “professional identit*” or “professional self concept” or “professional self-concept” or “professionals’ self-concept” or “inter-disc* identity” or “interdisc* identity” or “inter-profess* identity” or “interprofess* identity” or “multi-disc* identity” or “multidisc* identity” or “multi-profess* identity” or “multiprofess* identity” or “trans-disc* identity” or “transdisc* identity”) OR AB (“Dual identity” or “professional identit*” or “professional self concept” or “professional self-concept” or “professionals’ self-concept” or “inter-disc* identity” or “interdisc* identity” or “inter-profess* identity” or “interprofess* identity” or “multi-disc* identity” or “multidisc* identity” or “multi-profess* identity” or “multiprofess* identity” or “trans-disc* identity” or “transdisc* identity”)	1,176
2	TI (Checklist* or course* or educat* or guid* or intervention* or lesson* or model* or module* or prevent* or program* or “professional development” or resource* or strateg* or train* or workshop*) OR AB (Checklist* or course* or educat* or guid* or intervention* or lesson* or model* or module* or prevent* or program* or “professional development” or resource* or strateg* or train* or workshop*)	4,124,540
3	S1 AND S2	609

**Fig 1:**
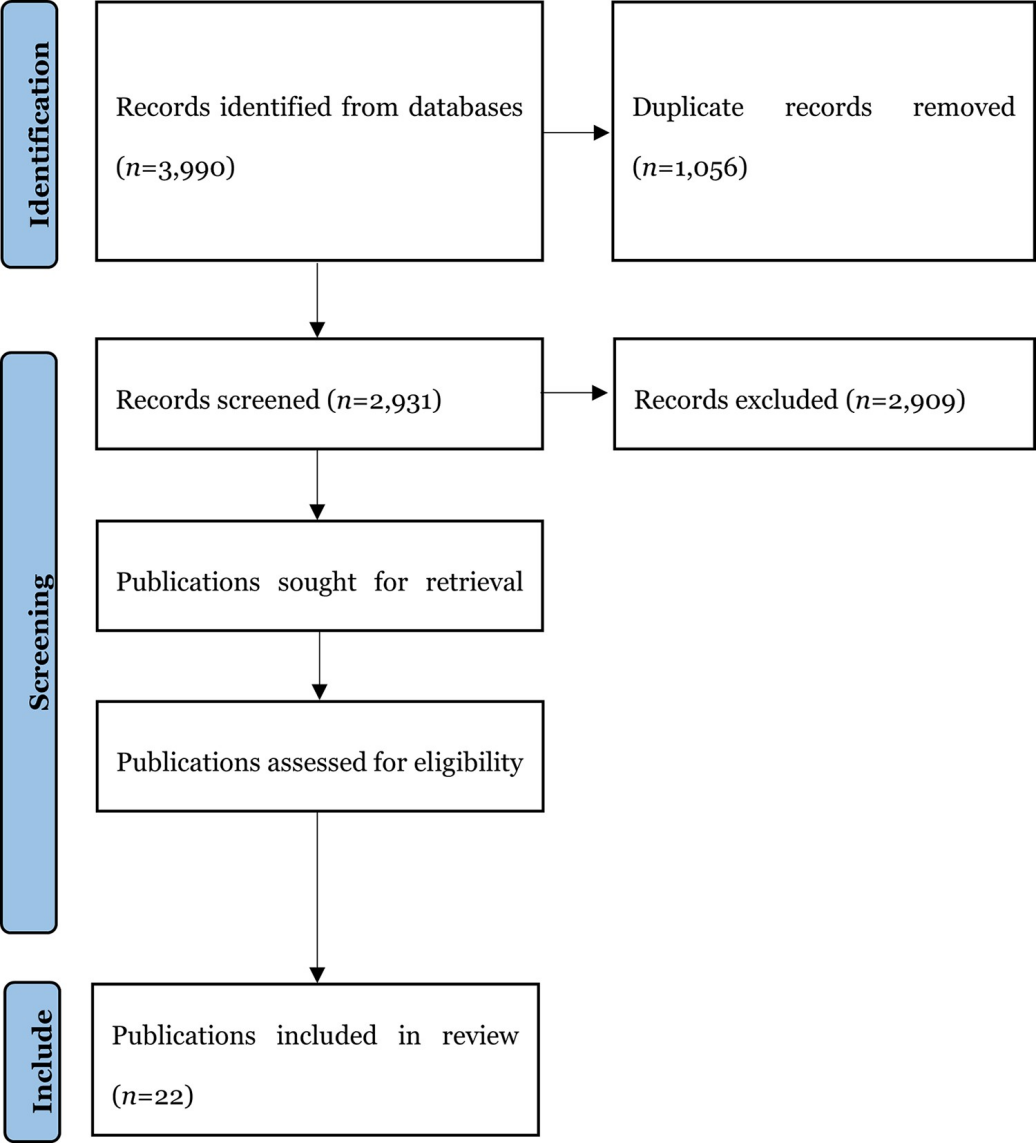
PRISMA flowchart [48].

Third, ten percent of the titles and abstracts were screened independently (AD and SB) with findings compared and discussed for rigour. A publication was excluded if it: was not published in English; represented a letter, a commentary, an editorial, or a book review; was devoid of an author; represented a systematic review or meta-analysis, given the limited detail typically presented about individual studies included in these publications; represented a protocol; represented a conceptual, theoretical, or methodological publication; was a descriptive publication, devoid of empirical findings (e.g., merely made a case for more research); included participants who were undergraduate or postgraduate students; or could not be sourced throughout Australian university libraries. Fourth, results were reported following the guidelines in a preferred reporting items for systematic reviews and meta-analyses (PRISMA-ScR) flow diagram [48] (see Fig 1). Following this, 22 publications warranted inclusion.

Fifth, content from the 22 publications was collated, summarised, and reported. Specifically, the following information was extracted and tabulated for narrative interpretation: the study location; the study aim; the setting; the population; the sampling method; the methodological approach; the intervention; its aim; key findings regarding professional identity; the author-identified limitations; and the author-identified future research opportunities. As a scoping (rather than a systematic) review, a critical appraisal of the publications was not warranted [25]. Data from five publications were extracted independently (AD, SB) and compared for accuracy before completing the data extraction process.

### Lexical review

To optimise the likelihood of a systematic approach to the lexical analysis [49], it was aided by Leximancer–data-mining software that uses Bayesian reasoning to detect key concepts and reveal their relationships [50]. Using algorithms, Leximancer identifies frequently occurring and co-occurring words and amalgamates these to form and visually map concepts that reflect themes within the text [51]. The maps convey three types of information–‘the main concepts in the text and their relative importance; the strengths of links between concepts (how often they co-occur); and similarities in contexts where links occur’ [52]. Concepts represent ‘collections of words that generally travel together throughout the text’ [53]. The components of these concepts are ordered within a thesaurus, comprised of relevant words and weightings to indicate relative importance. Within the map, connections between concepts that are most probable are represented by a spanning tree of grey lines or branches–specifically, ‘The spanning tree… shows the most-likely connections between concepts (like a road map of highways), but there are other (less-strong) connections between concepts (like backstreets)’ [54]. Clusters of concepts within a map–known as themes–suggest contextual similarity [55]. Themes are colour-coded to signify those that are (and are not) important, whereby the ‘most important theme appears in red, and the next hottest in orange, and so on according to the colour wheel’ [53].

In addition to the novelty of a lexical analysis using Leximancer, its value is threefold. First, Leximancer can manage a large corpus of qualitative data, akin to that sourced from publications. Second, relative to the other forms of computer-assisted qualitative data analysis software, like NVivo, the researcher is at a greater arm’s length of the analysis when using Leximancer–this can reduce researcher bias and increase reliability, as the researcher is encouraged to ‘discover’ [56, 57] how discourse travels together [58]. These reasons might partly explain growing interest in lexical analyses using Leximancer [59–63]. Third, although Leximancer has been used to methodically review literature in other fields–including infection control [64], knowledge management [65], marketing [66], nursing [67], and physical education [68]–it is yet to be used to ‘text mine’ literature on the mobilisation of professional identity. Leximancer was therefore used because it was fit-for-purpose.

Leximancer was used in three steps. First, once the text from each publication was collated (except the abstract, tables, figures, and references, thereby awarding focus to the prose within each publication), the ‘discovery’ mode was used to, ‘see what concepts were automatically generated by Leximancer without intervention’ [69]. Illustrating the automatically-generated relationships within the text, in the first instance, helps to ‘create learning and understanding’ [70] and identify ways to make sense of these relationships.

Second, Leximancer was used to examine the relationship between the concepts relevant to professional identity, and the concepts relevant to mobilisation. This involved creating two compound concepts. The first, ‘Professional identity’, was created by combining the concepts, ‘professional’, ‘professionals’, ‘identities’, ‘identity’, and ‘boundaries’, using the Boolean operator, ‘or’; while the second, ‘Mobilisation’, was created by combining the concepts, ‘change’, ‘develop’, ‘developed’, ‘development’, and ‘different’, also using the Boolean operator, ‘or’. The use of this Boolean operator ‘means that evidence for your compound concept will be calculated to include evidence for either of your concepts’ [71].

Third, for comparative value, each publication was associated with one tag. Tagging helps to compare the conceptual content of different data [54]. To determine whether (and how) study location influences the discourse within the publications, each publication was tagged according to one of four regional groupings–namely: the United States of America and Canada (*n* = 9); the United Kingdom (*n* = 6); Europe (*n* = 5), encompassing Croatia, Denmark, the Netherlands, Norway, and Portugal; and other (*n* = 2), encompassing China and Uganda. Once tagged, and guided by previous research [52], thirty concepts were profiled within each concept map to avoid diluting the focus of each map. To identify differences between locations, the thirty concepts were profiled using the themed discovery setting, ‘Concepts in Each’, to ‘discover concepts that distinguish… categories from one another’ [53]. For succinctness, attention is awarded to the word-like concept (rather than pronouns) that is most pertinent to each tag, as indicated by the likelihood percentage. Calculated by Leximancer, the likelihood percentage denotes the proportion of text segments that is shared by a tag concept and another concept, thus providing both directions of conditional probability [72].

## Results

### Scoping review

Of the 22 publications included in this review, five were published before 2010, five were published between 2010 and 2017, and 12 were published more recently from 2018 to 2022 (see Table 2); this suggests an ongoing interest in this field. Most of the studies reported in the publications were conducted in the United States of America (*n* = 8), six were conducted in the United Kingdom, and five, in European nations, suggesting a narrow geographical interested in this topic. Most of the publications pertained to healthcare (*n* = 15), with others set in school education (*n* = 3) and a range of administrative contexts (*n* = 3)–as such, caution is needed when interpreting findings across different workplace environments. Only three publications primarily reported on the collection and analysis of quantitative data.

**Table 2:** Scoping review summary.

Publication	Location	Setting	Aim	Population	Methods	Intervention	Intervention aim	Intervention effect on professional identity	Author-identified future research opportunities
Ball and colleagues [73]	United Kingdom	Healthcare education	Determine the impact and value of scenario-based learning	Postgraduate diploma pre-registration therapeutic radiographers, medical physics trainees, and radiation oncology registrars	Quantitative—pre- and post-event completion of the readiness for interprofessional learning scale and a Likert-style survey	Simulation, small working group practices and authentic scenario-based work-packages	Determine whether a simulated radiotherapy placement could improve mutual understanding of key roles among radiotherapy professionals and whether participants found it to be useful	Surveys pre and post intervention suggest an impact on professional identity, but does not account for pre intervention professional mutual respect	Investigations into how to foster more fruitful professional relationships between oncology professionals
Branch [74]	United States of America	Healthcare	Develop humanistic professional behaviours among faculty members (physicians)	Medical school faculty members and local facilitators	Quantitative–‘We measured feasibility and learner engagement by documenting a low dropout rate (in the range of 5% to 10%) over the year of the program and high attendance at the twice-monthly sessions (approximately 80%)’ [74]	Longitudinal faculty development programme	Improve humanistic teaching and role modelling	Analysis of participants’ personal narratives before and after the program indicated a change in personal identity	Advancement in professional identity formation over time using qualitative research
Byrnes and colleagues [15]	United States of America	Healthcare	Examine ways to change the perceived negative academic and cultural image of doctors, usually seen to be egotistical, paternalistic, and inflexible	Bariatric surgeons	Qualitative–interviews	Michigan bariatric surgery collaborative (MBSC) surgical coaching programme	Develop a coaching quality improvement programme based on the Wisconsin surgical coaching programme	Interviews with participants revealed the intervention had potential to influence professional identity	Explore surgeon identity and how socialisation occurs throughout a surgeon’s career
Chen and Reay [75]	United Kingdom	Public services organisations	Investigate how professionals respond when required to conduct work that does not match with their identity	Occupational therapists, occupational therapist assistants, and caseworkers	Qualitative–interviews	Major job redesign initiative, including role and title	Improve support for the frail elderly population and thus allow them to remain in their home through more integrated health and social care	Semi-structured interviews and documentary analysis, pre intervention and one year later, showed many people still resisted identity change, mourning the loss of previous work; those who recognised value in the new way of working appeared to modify their previous professional identity	Explore how the increasing multidisciplinarity of professions is interpreted, responded to, and negotiated by professionals
Croft and colleagues [76]	United Kingdom	Healthcare	Explore how attachment to professional identity could result to identity conflict	Nurse managers undergoing a leadership development programme	Qualitative–interviews and observations	Two leadership development programmes for nurses moving into roles requiring the construction of both nurse and leader identities	Ease transition into roles requiring the construction of both nurse and leader identities	Observation (one researcher enrolled with the course) with semi-structured interviews showed how desired group identities can undermine the construction of leaders’ identity	Identify ways people could emotionally detach themselves from their ability to construct a desired social group identity
Currie and colleagues [77]	United Kingdom	Healthcare	Explore human resource practices that influence doctors’ knowledge-brokering to improve service delivery	Metro leadership team and doctors enacting a knowledge brokering role	Qualitative–interviews and observations	Human resource practices, such as performance management, training and development, and job design	Influence of human resource practices on enactment of doctors’ knowledge-brokering role to drive service improvement in healthcare	Some human resources practices can stifle professional identity (e.g., performance management), while others can promote its development (e.g., aligning job title and position description with the role)	Explore knowledge transition in public sector organisations among professionals
Dehn and colleagues [78]	Denmark	Healthcare	Investigate nurse experiences of learning and guided self-determination (GSD) in clinical practice and explore whether, and potentially how GSD contributes to a renewed long-term understanding of their professional role and identity	Nurses educated in GSD in three gynaecological settings	Qualitative–interviews	Education in GSD	Support patients to develop life skills to cope with their condition	Participant interviews indicated the importance of others in shaping professional identity when undertaking a new role	Not reported
Gottlieb and colleagues [79]	Canada	Medical education	Describe the implementation of the advanced research methodology evaluation and design in medical education (ARMED MedEd) course	Clinician educators	Quantitative–survey including open-ended feedback	Longitudinal education programme	Address the need for advanced training in education research	An online mixed-methods survey of participants at baseline and post-course completion participants reported a substantial impact on their professional identity	Not reported
Heldal and colleagues [80]	Norway	Healthcare	Examine how human resource practices can resolve issues about professional legitimacy and identity and ensure improvement in professional practices	Nurses and hospital managers	Qualitative–interviews and observations	Patient safety programme	Reduce patient harm, build sustainable structures for patient safety, and improve the patient safety culture	Observation, and group and individual interviews indicated that the intervention reconstructed participants’ professional identity	Research the significance of the changes arising from patient-nurse relationships
Hennein and colleagues [81]	Uganda	Healthcare	Identify the communities of practice components that are core to behaviour change	Community health workers	Qualitative—interviews	Community of practice	Improve tuberculosis contact investigation	Interviews informed by behaviour change theory showed the intervention developed both social and professional identity	Explore the extent to which outcomes of a community of practice are due to the community of practice or arise from the facilitation of external parties
Hu and colleagues [82]	China	Healthcare	Investigate the effect of a transition programme including a cognitive-behaviour-based preceptorship intervention on ICU new graduate nurse professional identity and their intention to remain employed	ICU nurses–new graduates	Quantitative—survey	Transition programme	Enable nurses to develop their thinking and skills to cope with problems in their professional work through cognitive restructuring	Survey data from baseline and post intervention demonstrated an impact on professional identity; though at six-month follow-up, this had declined	Investigate staff members’ expectations and attitudes to interventions addressing the mobilisation of professional identity, including longitudinal studies with larger groups
Jordan and colleagues [83]	United States of America	Healthcare	Explore the impact of medical education fellowships on the careers of graduates	Medical graduates	Qualitative—interviews	Medical education fellowship	Provide professional development, protected time, dedicated mentorship, and continued clinical practice	Semi structured interviews showed the intervention cemented participant professional identity or facilitated their identity to evolve	Not reported
Lifshitz-Assaf [84]	United States of America	Administration	Examine the role of open/peer-production, innovation among research and development professionals and their services	Research and development professionals	Qualitative–interviews, observations, and document analysis	Open innovation model	Elevate innovation performance	Despite initial resistance, observations, interviews, and analysis of project documents, before and after the intervention, showed the professional identity of those who adopted open innovation was impacted; professional identity of those who continued to reject the intervention remained unchanged	Not reported
Lopes [85]	Portugal	Education	Evaluate changes in professional identity resulting from curriculum development	Teachers	Qualitative–fieldnotes, tape recordings, and questionnaires	Curriculum development	Ascertain how curriculum development can promote professional identity	Field notes and questionnaires at baseline and on completion of the intervention, plus focus groups, showed professional identity was impacted, though not always in the same way	Investigate professional focus on the ‘why’ of their work, rather than ‘how’, which changed as their disciplines and projects changed
Ludden and colleagues [86]	United States of America	Healthcare	Examine issues of concern, such as professional identity, relationships with work colleagues, and problems associated with handling patients to members	Physicians and nurses	Qualitative–observation	Loosely structured seminar series, including lunch–five groups meeting weekly for ten months	Prepare primary care staff for psychological aspects of primary care	Observations showed the intervention facilitated the establishment and redefinition of professional identities in relation to each other	Not reported
Luehmann [87]	United States of America	Education	Explore the role of a blog in improving professional identity	Teachers	Qualitative–single case study	Blog	Support development of professional identity and reflect on professional practice and engagement with a professional community	Text analysis, email exchanges, and interviews with other colleagues revealed the shift in professional identity over the life of the intervention	Not reported
Luke and Gordon [88]	United States of America	Education	Improve medical professionals’ professional identity	School counselling interns	Qualitative–document analysis	Supervision by email	Enhance computer-mediated supervision and provide more access to supervision for school counsellors using computer-mediated supervision	Observations and analysis of supervisory email texts indicated a shift in professional identity during the intervention	Not reported
Malin and Morrow [89]	United Kingdom	Healthcare	Provide a platform for family bonding with children	Children centre team members	Qualitative–interviews	Development programme	Examine the use of interprofessional work in a child centre through a development programme	Interviews indicated how the intervention impacted professional identity, though there was some reluctance	Research on how supervisee and supervisor email discussions can change over time with respect to others
Molleman and colleagues [90]	Netherlands	Healthcare	Investigate the consequence of multidisciplinary medical team meetings for surgical and medical specialists	Medical specialists	Quantitative–survey	Participation in a multidisciplinary medical team meeting	Allow physicians from different medical specialties to regularly meet to discuss patients and engage in collaborative decisions	Participant survey showed the intervention had little impact professional identity	Not reported
Netting and Williams [91]	United States of America	Healthcare	Examine the implications of changing roles and relationship between case managers and doctors on professional identity	Doctors and case managers	Qualitative–interviews	Generalist physician initiative	Improve collaboration between case managers and doctors to improve primary care of elderly people	Interviews at all intervention sites indicated challenges for professional identity as a result of the intervention, particularly re others’ perceptions	Research on the impact of informal ‘backstage’ interactions
Nichol and Williams [92]	United Kingdom	Administration	Examine the interrelationship between practitioners’ own understanding of professional identity and that which is embedded within the professional body	Human resources staff	Qualitative–interviews	Work-based learning programme	Close the gap between professions’ own narrative of professional identity and the that of the professional body of his/her profession	Interviews revealed the intervention did not influence professional identity, but the workplace environment impacted the development of a professional identity unique to each participant	Not reported
Wolf and colleagues [93]	Croatia	Administration	Clarify how practitioners in public employment services cope with continuous growth demand	Public service employees	Quantitative–self-assessment questionnaire	Peer coaching	‘strengthen [practitioners’] skills in facilitating solution-finding at the workplace with their colleagues and to enrich their support of clients by approaches like powerful questioning, active listening and mind growth’ [93]	A self-assessment questionnaire, pre and post intervention, suggests the intervention can transform professional identity	Not reported

#### Supporting the mobilisation of professional identity

Twenty-two unique interventions were identified that can mobilise professional identity. However, not all were explicitly targeted at professional identity, with nine publications stating a focus on professional identity in either the study aim, or intervention aim [75, 76, 78, 82, 85–87, 91, 92]. The most common intervention was a training or development programme (*n* = 11) [15, 73, 74, 76, 78–80, 83, 86–88], followed by a change in workplace design (*n* = 8) [75, 82, 89–93]–these included, for instance, the impact of curriculum development [85]. Other interventions included a blog of a single teacher, suggesting limited opportunity to generalise from the findings [87], peer coaching [93], and participation in a community of practice [81]. Most of the training and development programmes focused on the professionals’ development [15, 73, 76, 78, 79, 83, 86, 91, 92]–for instance, Branch [74] developed a faculty development programme to promote humanistic professional behaviours among faculty members–namely, physicians. However, two publications were more directly focused on patients via, for example, a patient safety programme [80, 89]. Wolf and colleagues [93] noted that transforming professional identity is not a one-off event–but rather, ongoing efforts are required to support professionals during the transformational process. Approaches to assess intervention effect on professional identity varied. Most publications reported on qualitative methods [15, 76, 78, 81, 83, 86, 89, 91, 92], with others using mixed methods [75, 77, 79, 80, 84, 85, 87, 88], and the remaining five leaning on quantitative methods [73, 74, 82, 90, 93]. Most of the interventions were longitudinal, with much of the data collection using a longitudinal approach to evaluate the impact of the intervention on professional identity [73–77, 79, 82, 84–88, 93]. Others drew one timepoint, using–for instance–single interviews, limiting the potential to gauge impact over time. Most studies reported that the intervention influenced professional identity [15, 74, 76–81, 83, 85–89, 93]. For example, Heldal and colleagues [80] stated that the intervention reconstructed participants’ professional identity. Yet two publications reported the intervention did not shift professional identity [90, 92], and five indicated that the intervention had impacted professional identity with a caveat [73, 75, 82, 84, 91]. For example, Hu and colleagues [82] reported the intervention impacted professional identity, though impact waned at six months, post intervention; similarly, Ball and colleagues [73] qualified the positive findings, noting that the results did not account for participants’ pre intervention mutual respect.

While all the publications discussed approaches to transform professional identity, several identified specific considerations. For example, Croft and colleagues [76] explored how nurses who moved into management positions managed their identity conflict. They reported three choices–namely: reaffirming their nurse professional identity while distancing themselves from the leader identity; framing their leader identity with the language associated with their nurse identity; or overcoming their attachment to the nurse identity and engaging with the management identity–the latter of which demands eschewing rather than mobilising professional identity. Luke and Gordon [88] examined supervision by email, offering practical and potentially transferable suggestions to transform identity–these included the careful selection of words and phrases (e.g., professional jargon) and using plural nouns to create shared alignments as part of the same professional community.

Several authors recognised the need to clarify roles and identify boundaries to facilitate a shift in professional identity [73, 75, 78, 83, 84, 89, 91]. This was of particular importance when professionals were required to blur their boundaries. Consider, for instance, the Sure Start programme–a multi-professional early childhood initiative in the United Kingdom–where professionals assumed transdisciplinary roles. However, this blurring of boundaries required support through supervision.

#### Drivers to mobilise professional identity

Several authors reported the impetus or drive to mobilise professional identity. They raised the challenge of identity conflict [15], which can be triggered by: a role change–for instance, from clinician to manager [76]; extending the scope of practice, as demonstrated by incorporating mental healthcare in primary care [86]; or an intervention, like peer coaching [93] or workplace changes [85].

Most of the interventions provided an external driver–that is, the demands or expectations of others–to shift professional identity [15, 74, 75, 78, 82, 85, 86, 88–90, 93]. Two publications suggested this approach can help to flex professional identity by raising awareness of a role; though it was unclear who to [80, 91]. Malin and Morrow [89] stressed the significance of the supervisors’ role to blur roles and boundaries, while Ludden and colleagues [86] as well as Nichol and Williams [92] were clear that co-worker interactions had the greatest influence on professional identity.

Other authors, however, indicated that the professional required internal motivation to initiate and drive a change in professional identity [76, 84]. For example, Luehmann [87] described the novel approach of blogging to interrogate the professional identity of an American school teacher to ‘unpack’ different components influencing professional identity.

While most of the publications referred to an external or an internal driver to mobilise professional identity, Jordan and colleagues [83] spoke of the complementary value of both. While they recognised a fellowship programme as an external driver, they also recognised participants’ personal interests in their field (here, medical education) as an internal driver, offering participants the opportunity to ‘recognise their own personal niche’.

### Lexical review

**Fig 2:**
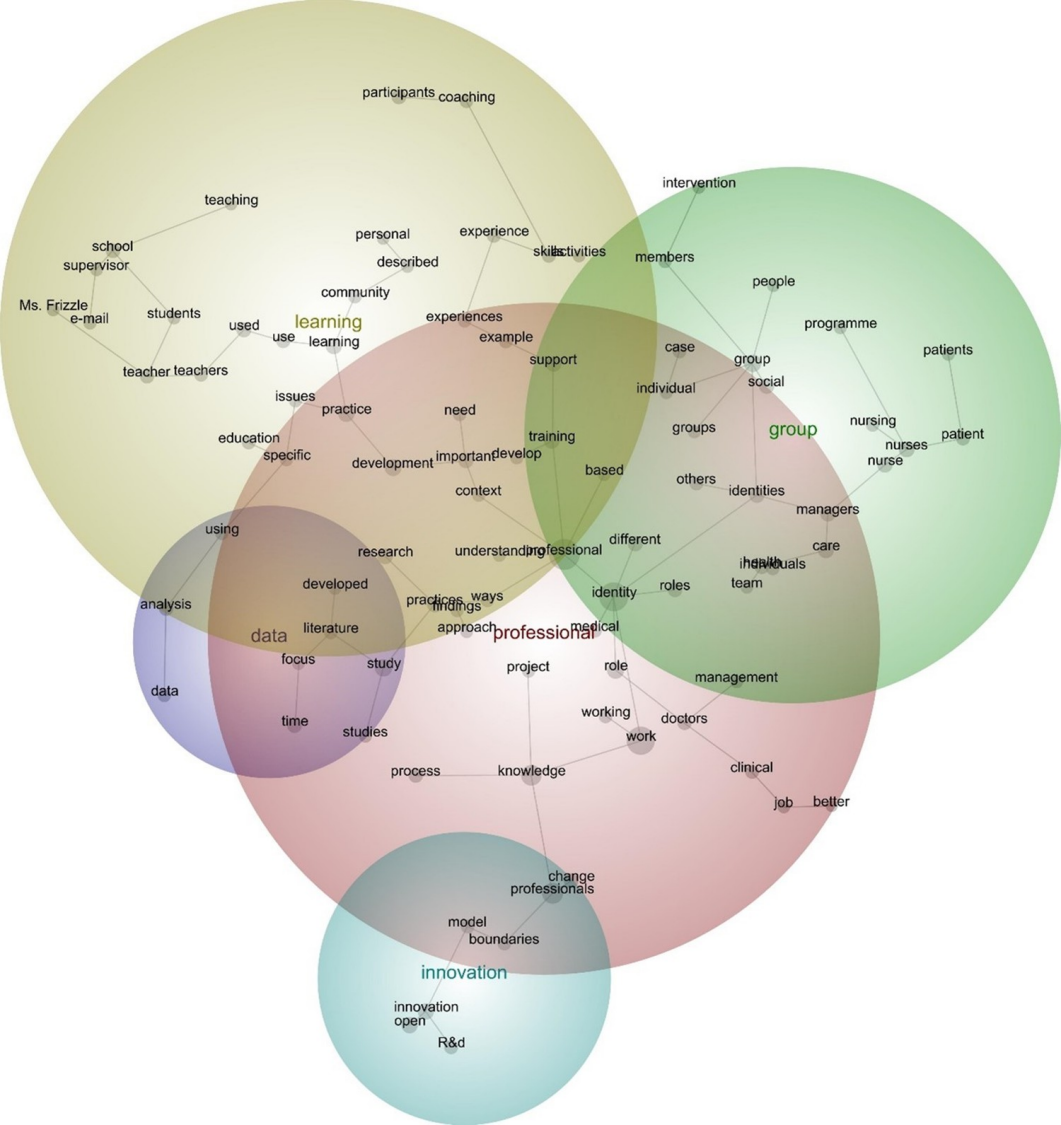
Discovery mode concept map (visible concepts: 100%, theme size: 60%)^1^. ^1^ Colour code: red denotes the most important theme, followed by yellow, green, aqua, and purple.

The concept map and the thematic summary revealed five themes–namely: ‘professional’, ‘learning’, ‘groups’, ‘innovation’, and ‘data’ (see Fig 2). These highlight the key clusters of concepts, or topics, represented within the text of the publications. Theme position illustrates the relationships between the themes. Consider for instance, the prominence of ‘professional’, which appeared in red and overlapped with the four remaining themes, particularly ‘learning’. This suggests that when the publications referred to ‘professionals’ (and the concepts therein), they were inclined to refer to ‘learning’ (and the concepts therein)–and given the distance between the themes, ‘learning’ and ‘innovation’, when the publications referred to the former (and the concepts therein), they were not inclined to refer to latter (and the concepts therein):

The participants identified that through undertaking work based learning they evaluated their professional practice formed in the organisational context, against the theoretical models of HR and the professional body’s standards thereby self-validating their own practice. Often the participants wanted to demonstrate that their expertise was comparable with their colleagues who had studied through the conventional pre-work experience route [92].

The innovation lead described the sense of fear and discomfort in the room that day: When you asked [whether you have an R&D problem to share], they’d say, ‘‘You want me to tell you what I can’t solve?” It was very much like they would be exposing some kind of incompetency if they told us what they can’t do [84].

**Fig 3:**
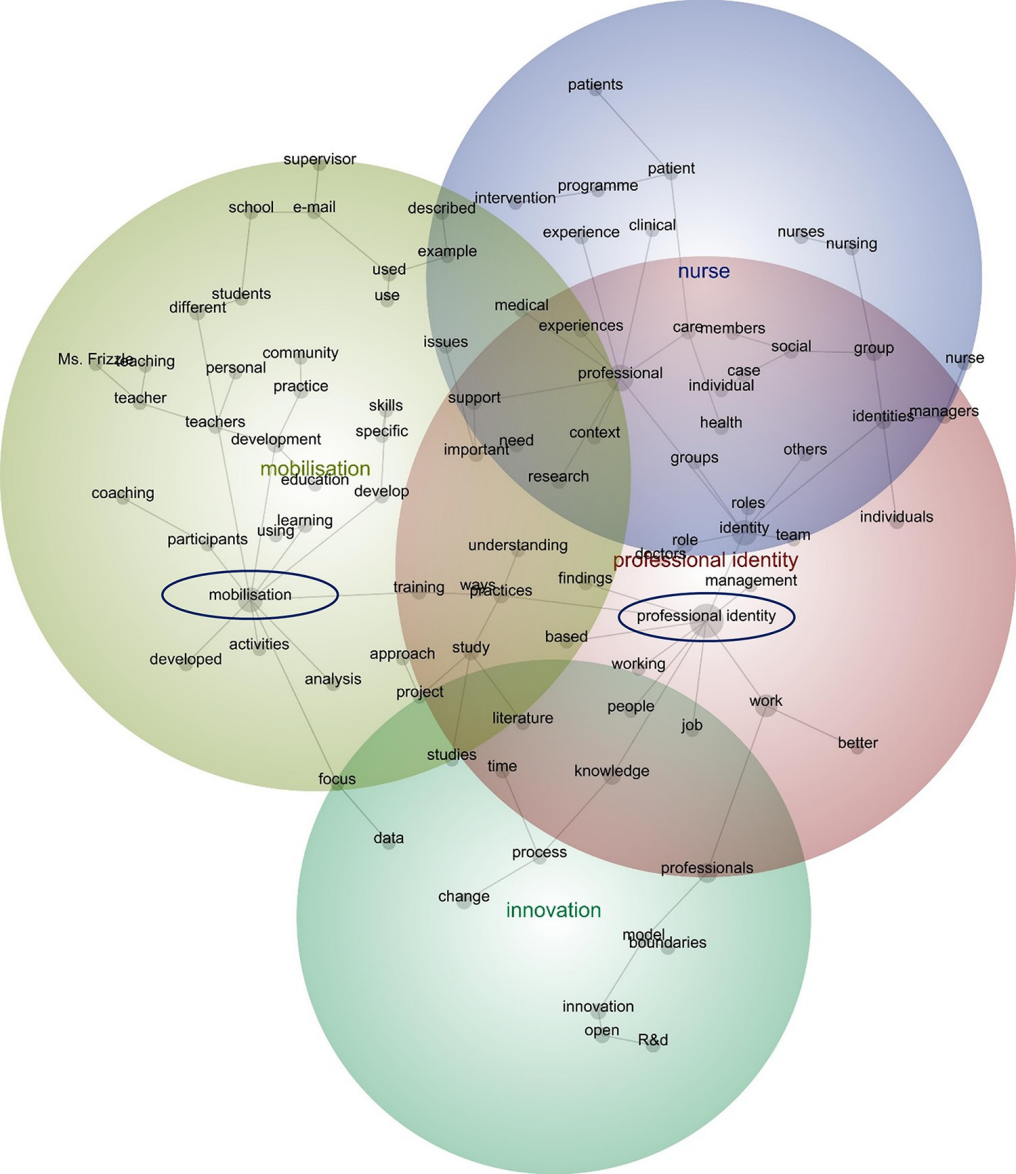
Concept map after creating compound concepts (visible concepts: 100%, theme size: 60%)^1^. ^1^ Colour code: red denotes the most important theme, followed by yellow, green, and purple.

Given their relevance to this review, the relationship between the two compound concepts–‘Professional identity’ and ‘Mobilisation’–was examined. The grey lines or branches between the two illustrate a probable connection or relationship (see Fig 3). This was affirmed by the likelihood percentage, which suggests that over half (58%) of the text segments containing the concept, ‘Professional identity’, also contained the concept, ‘Mobilisation’ (see Table 3):

Compared with interdisciplinary teams, teamwork among physicians has been much less investigated. As we will argue, it is quite possible that different specialties have distinct professional identities and that this may lead them to perceive the consequences of teamwork differently [90].

**Table 3:** Word-like concepts connected with ‘professional identity’ (conditional probability > 55%).

Concept	Co-count	Likelihood (%)^1^	Concept	Co-count	Likelihood (%)^1^
professional	715	100	studies	41	80
identity	631	100	education	44	80
professionals	372	100	research	86	80
identities	140	100	care	82	80
boundaries	92	100	learning	78	80
supervisor	46	98	doctors	62	79
nurse	106	97	need	50	79
managers	112	96	working	61	79
model	84	95	personal	38	79
open	149	94	approach	34	79
individuals	79	94	members	41	79
work	453	94	study	119	79
individual	38	93	focus	35	78
e-mail	49	92	clinical	58	77
innovation	151	92	job	40	77
process	95	90	practice	92	77
findings	56	90	training	81	76
nursing	55	90	medical	48	76
group	151	89	analysis	51	76
role	140	89	issues	35	76
experiences	45	88	better	25	76
time	79	88	school	59	76
ways	69	87	patient	49	74
literature	48	87	teachers	40	74
used	67	87	community	36	73
nurses	94	86	skills	49	73
roles	80	86	coaching	40	73
case	55	86	example	42	72
context	54	86	support	67	72
others	69	85	team	23	72
experience	67	85	specific	30	71
knowledge	193	84	data	37	71
understanding	52	84	using	31	70
based	45	83	project	36	69
people	40	83	participants	58	69
practices	104	83	students	30	68
programme	42	82	change	114	67
social	91	82	management	30	67
described	36	82	development	133	66
important	76	82	activities	32	65
use	55	81	patients	30	62
intervention	42	81	teacher	44	62
health	67	81	develop	46	60
groups	46	81	mobilisation	375	58

^1^ A measure of the direct one-way affinity between two concepts, calculated by the fraction of times ‘Professional identity’ is coded of the total number of times each tabulated concept is coded.

The two compound concepts–‘Professional identity’ and ‘Mobilisation’–were connected by the concepts, ‘training’ and ‘practices’. This suggests that when the publications spoke of ‘Professional identity’, the discourse traversed ‘training’ and ‘practices’ when discussing ‘Mobilisation’, and vice versa:

A distinctive professional identity is developed and shared among a professional group through enhanced career prospects, socialization, long and rigorous training, and a lifetime spent doing the same tasks with a group of peers (Pratt, Rockmann, & Kaufmann, 2006). At the core of professional identity are moral values that underpin a sentiment of care for the client a professional serves, which transcend self-interest or organizational interest (Brint, 2015; Wright, Zammuto, & Liesch, 2017) [77].

However, this is not to overstate the connection between the two compound concepts, as ‘Professional identity’ was more likely to travel will other concepts. Notwithstanding those for which a strong relationship would be expected, like the concepts that were compounded–namely, ‘professional’, ‘professionals’, ‘identities’, ‘identity’, and ‘boundaries’–the concept, ‘Professional identity’ was highly likely to travel with discourse pertaining to ‘supervisor’, ‘nurse’, and ‘managers’. While the concepts, ‘supervisor’ and ‘managers’ spoke of their roles in shaping professional identity, discourse about ‘nurse’ largely referred to efforts to clarify if not retain a particular professional identity:

Professional Identity Physicians’ Perspectives. Physicians discussed the functions performed by their case managers and why a particular professional had been selected for the position [91].

The nurse explained that documentation was even more vital after the introduction of the patient safety programme and that it is ‘proof’ that the required work has been performed. It is not enough to conduct patient safety work or to say that it has been carried out (trust in professionals): it has to be documented (trust in systems) [80].

These findings suggest that the relationship between the discourses pertaining to ‘Professional identity’ and ‘Mobilisation’ was not a prominent feature among the publications. But rather, when the publications referred to ‘Professional identity’, they spoke of other concepts.

**Fig 4:**
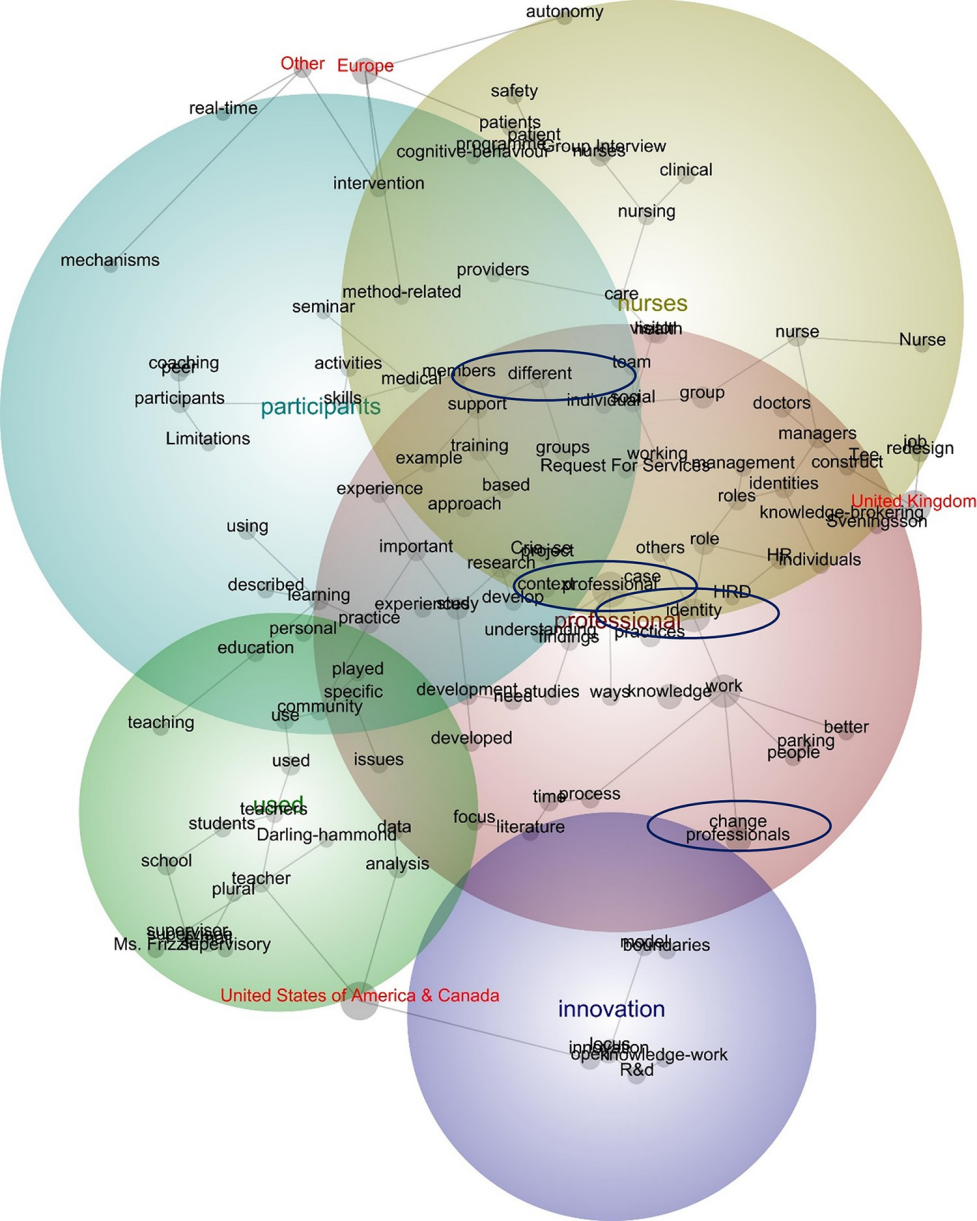
Concept map tagged by geography (visible concepts: 100%, theme size: 60%)^1^. ^1^ Colour code: red denotes the most important theme, followed by green and blue.

The 22 publications reported on studies conducted in the United States of America and Canada (*n* = 9), the United Kingdom (*n* = 6), Europe (*n* = 5), and other nations (*n* = 2; see Fig 4). As indicated by the spanning tree, publications from the United States of America and Canada were connected with discourse pertaining to ‘teachers’, ‘analysis’, and a cluster of concepts pertaining to ‘innovation’. Publications from the United Kingdom were connected with discourse pertaining to ‘redesign’, ‘construct, and ‘knowledge-brokering’. European publications were connected with discourse pertaining to ‘autonomy’, ‘patients’, ‘method-related’, and ‘intervention’; while publications from the remaining nations spoke of ‘intervention’, ‘mechanisms’, and ‘real-time’. Given the position of the tags that denote region, these findings suggest two points. First, the discourse presented in publications from Europe and the remaining nations–namely, China and Uganda–were largely similar. Second, the discourse presented in publications from United States of America and Canada, the United Kingdom, as well as Europe and the other nations differed–that is, while all the publications spoke of professional identity, they differed by region on how they did this:

Opening work boundaries challenges professionals’ identity, and the ability to reconstruct and refocus that identity becomes a critical mechanism for knowledge-boundary work and shifting the locus of innovation. Without ‘‘identity work,” there may be no true change in the knowledge work of professionals who might otherwise appear to adopt the open model (84, publication from the United States of America).

Calling clients beforehand helped professionals accomplish the new task—the financial assessment, with new and creative routines that did not exist prior to the job redesign (75, publication from the United Kingdom).

This provided the learners with some autonomy around their learning, so that they could choose for themselves how deep, how often and how quick they explored the learning content provided. Berninger-Schaefer (2012) introduces a similar way of developing peer coaches where the participants gain knowledge on peer coaching in addition to a one-day workshop for practice (93, publication from Europe).Almost 35% to 60% of nurses left their first workplace within the first year of hiring (Flinkman & Salanterä, 2015; Van Camp & Chappy, 2017; Zhang et al., 2017). However, none of the ICU NGNs left their positions within 18 months after the intervention (82, publication from China).

Given the focus of this review, the relationships between the study location and the concepts, ‘professional’, ‘identity’, ‘change’, and ‘different’ (which were not created as compound concepts) were considered. As indicated by the likelihood percentages (see Table 4), publications from the United Kingdom were relatively more likely to travel with discourse pertaining to the concepts, ‘professional’ and ‘identity’, while publications from the United Kingdom as well as United States of America and Canada were relatively more likely to travel with discourse pertaining to the concept, ‘change’. Conversely, notwithstanding the concept of ‘different’ in the European publications, the four concepts of interest did not feature prominently in the publications from Europe or the other nations:

When boundaries of distinct groups appear to come under threat from integration of various professional groups, there may be a sense of loss of professional identity, there may be tribalism or feeling of ambivalence, conflict and grief, and the need to maintain the self-confidence of different professional groups is very real (Atkins, 1998). The problem with trying to create professional partnership, according to Sennett (89, publication from the United Kingdom).

Our research asserts that it is critical to understand occupational organization and culture when attempting to create structural or policy change and intentionally design spaces for change. Overwhelmingly, surgeons in this study felt comfortable addressing their vulnerability and engaging in public quality improvement because of their connection to a professional organization (15, publication from the United States of America).

**Table 4:** Likelihood percentage between location tag and relevant concepts.

Study Location	Concept	Co-count	Likelihood (%)
United States of America and Canada	• ‘professional’	242	34
	• ‘identity’	196	31
	• ‘change’	67	39
	• ‘different’	49	27
United Kingdom	• ‘professional’	288	40
	• ‘identity’	311	49
	• ‘change’	66	39
	• ‘different’	63	34
Europe	• ‘professional’	138	19
	• ‘identity’	91	14
	• ‘change’	31	18
	• ‘different’	62	34
Other nations	• ‘professional’	47	7
	• ‘identity’	33	5
	• ‘change’	6	4
	• ‘different’	9	5

## Discussion

For personal, social, and economic reasons, interprofessional healthcare makes sense, offering patients and carers timely access to appropriate healthcare. This is particularly important given the prevalence of chronic and complex illnesses [5, 6], and the associated implications for resource-limited health services [9–11].

Despite the aforesaid (and potentially) other benefits, interprofessional healthcare can be difficult to enact and demonstrate, particularly that which is effective. This is not to suggest an absence of research. For instance, a series of publications highlighted the importance of: collaboration; resource pooling; learning; role blurring; communication; influence; behavioural norms; a shared purpose; critical reflection; innovation; and leadership [94–97]. These and other studies are informative [98], helping to affirm that interprofessional healthcare ‘goes beyond good camaraderie’ [99]. However, they do little to clarify the role of professional identity in interprofessional healthcare, and how it might be intentionally primed for such care. Although one can source ideas and suggestions, there appears to be limited, if any empirical support for these [42, 100].

This review clarified, empirically, that the professional identity of established professions can be mobilised [15, 73–76, 78–89, 91, 93], whether or not this was the primary intention of the intervention. This was principally achieved via a training or development programme for professionals and/or patients [15, 74] and, to a lesser extent, a change in workplace design [85], a blog [87], and peer coaching [93]. Although the primary intention of the interventions in relation to professional identity appeared at times to be ad hoc, several design features were paramount. Central to the design of many of the interventions was their longitudinal nature, suggesting it is unlikely for professional identity to be mobilised via single initiatives. Additional design features included the role of a driver–the stimulus to impel mobilisation. While these were predominately external–like a role change or an intervention [86]–the role of internal motivation, whereby participants were open to change, was also recognised [84].

Complementing the findings from the scoping review, the lexical review was also informative, highlighting two key findings. First, within the publications, the relationship between professional identity and mobilisation did not feature prominently–as such, the discourse pertaining to the former did not travel closely with that pertaining to the latter. Second, geography matters–while publications from the United States of America and Canada were more likely to feature discourse pertaining to, for instance, innovation, those from the United Kingdom were more likely to highlight discourse about, for instance, knowledge-brokering. Similarly, while European publications were more likely to feature discourse pertaining to, for instance, patients and autonomy, those from China and Uganda were more likely to highlight discourse about, for instance, mechanisms. Although the reasons for these findings are beyond the scope of this study, they open opportunities for further research.

Despite the value of the aforesaid findings, the search strategy might have failed to identify all relevant publications. In addition to the use of a focused search strategy, this is because of the myriad terms for professional identity and ways to ignite mobilisation. For instance, although potentially relevant, a study on ‘the reconstruction of professional role identity’ (which was identified when developing and testing the search strategy) was not identified via the search strategy deployed in this study [101]. Furthermore, this publication also highlights the diffuse ways in which an ‘intervention’ is defined. For instance, as a naturalistic study on the restructure of a health clinic, it might have been excluded from this review. However, the authors also spoke of the organic nature of the ‘intervention’, whereby–‘The changes we studied were initiated by the clinic physicians themselves’. This suggests that reviews that are more encompassing would require clear criteria to determine which of the publications that report on naturalistic studies warrant inclusion.

## Conclusions

Notwithstanding the aforesaid limitation, this article has considerable implications for scholars and practitioners. For scholars, there is the customary call for more research–however, what is specifically required is clarity in five areas. The first pertains to the facets of professional identity and how these might be gauged. The second regards the types of interventions that are more likely to prime professional identity for interprofessional care, with reference (but not limited) to: the target audience(s) or profession(s); content; mode and frequency of delivery (or exposure); duration; as well as setting or context. Relatedly–and reflecting others’ advice [76, 81]–a third area of research is that which serves to elucidate how exactly professional identity was changed by an intervention and the conditions that support and sustain the change. Given the lexical review, the fourth is research to clarify contextual differences in the field of research and the associated reasons–this reflects others’ call for research that accounts for organisational and/or professional context [15, 77]. The fifth, and perhaps most important area for research, is the impact associated with interventions that prime professional identity for interprofessional care. This encompasses the outcomes at: a personal level–like those experienced by professionals, patients, and carers; a social level–like those demonstrated within a team, an organisation, and a community or network [19]; and an economic level.

For practitioners, particularly managers and those affiliated with professional bodies, the implications associated with this article are threefold. First, regarding the practical applicability of the interventions, although the 22 publications included limited detail on the resources required for the interventions, the demand on resources varied widely. For instance, there were references to a major job redesign initiative [75], a loosely structured seminar series [86], supervision by email [88], and a blog [87], among others. This suggests there is considerable scope for practitioners to determine the feasibility of these interventions relative to their anticipated value.

Second, despite the demonstrated benefits of interprofessional care, there is very limited evidence to guide how professional identities might be primed and shaped to enable, promote, and sustain such care. As such, caution is warranted when implementing interprofessional care programs.

Third (and relatedly), this limited evidence opens opportunities for collaborative and impactful inquiry between practitioners and scholars. Managers and those affiliated with professional bodies for practitioners have a responsibility to prepare and support their staff and members, respectively, for interprofessional practice. As such, engaging with health service management scholars might serve to clarify how to purposely shape professional identities towards this aim.

## Supporting information

S1 Checklist Preferred Reporting Items for Systematic reviews and Meta-Analyses extension for Scoping Reviews (PRISMA-ScR) checklist. (DOCX)
